# Immunofluorescence analyses of respiratory epithelial cells aid the diagnosis of nephronophthisis

**DOI:** 10.1007/s00467-024-06443-0

**Published:** 2024-08-05

**Authors:** Carlotta Hellmann, Kai Wohlgemuth, Petra Pennekamp, Sebastian George, Mareike Dahmer-Heath, Martin Konrad, Heymut Omran, Jens König, for the NEOCYST Consortium, C. Bergmann, M. Cetiner, J. Drube, C. Gimpel, J. Göbel, D. Haffner, T. Illig, N. Klopp, M. C. Liebau, S. Lienkamp, C. Okorn, L. Pape, F. Schaefer, B. Schermer, H. Storf, A. Titieni, S. Weber, W. Ziegler, I. Kamp-Becker, J. Vasseur, S. Kollmann, J. Gerß

**Affiliations:** Department of General Pediatrics, University Children’s Hospital Münster, Albert-Schweitzer-Campus 1, 48149 Münster, Germany

**Keywords:** Nephronophthisis, Renal ciliopathies, Immunofluorescence, NPHP1, NPHP4, Transition zone

## Abstract

**Background:**

Nephronophthisis (NPH) comprises a heterogeneous group of inherited renal ciliopathies clinically characterized by progressive kidney failure. So far, definite diagnosis is based on molecular testing only. Here, we studied the feasibility of NPHP1 and NPHP4 immunostaining of nasal epithelial cells to secure and accelerate the diagnosis of NPH.

**Methods:**

Samples of 86 individuals with genetically determined renal ciliopathies were analyzed for NPHP1 localization using immunofluorescence microscopy (IF). A sub-cohort of 35 individuals was also analyzed for NPHP4 localization. Western blotting was performed to confirm IF results.

**Results:**

NPHP1 and NPHP4 were both absent in all individuals with disease-causing *NPHP1* variants including one with a homozygous missense variant (c.1027G > A; p.Gly343Arg) formerly classified as a “variant of unknown significance.” In individuals with an *NPHP4* genotype, we observed a complete absence of NPHP4 while NPHP1 was severely reduced. IF results were confirmed by immunoblotting. Variants in other genes related to renal ciliopathies did not show any impact on NPHP1/NPHP4 expression. Aberrant immunostaining in two genetically unsolved individuals gave rise for a further genetic workup resulting in a genetic diagnosis for both with disease-causing variants in *NPHP1* and *NPHP4*, respectively.

**Conclusions:**

IF of patient-derived respiratory epithelial cells may help to secure and accelerate the diagnosis of nephronophthisis—both by verifying inconclusive genetic results and by stratifying genetic diagnostic approaches. Furthermore, we provide in vivo evidence for the interaction of NPHP1 and NPHP4 in a functional module.

**Graphical abstract:**

**Figure:**
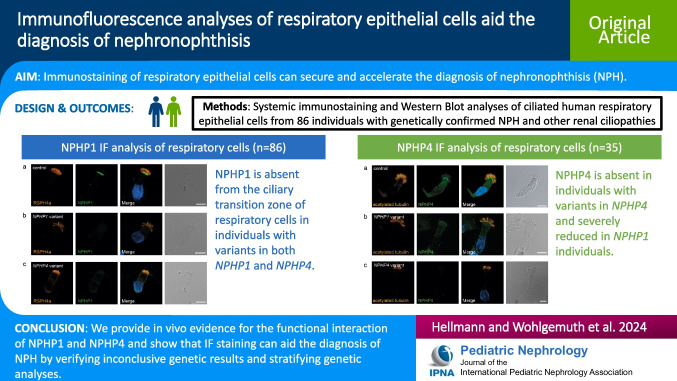
A higher-resolution version of the Graphical abstract is available as Supplementary information

**Supplementary Information:**

The online version contains supplementary material available at 10.1007/s00467-024-06443-0.

## Introduction

Renal ciliopathies comprise a heterogeneous group of rare hereditary kidney diseases frequently characterized by a slowly progressive loss of kidney function and the presence of kidney cysts. Although rare individually, as a group they represent one of the most frequent causes of kidney failure in childhood and adolescence. The main representatives of this group are nephronophthisis (NPH) and nephronophthisis-related ciliopathies (NPH-RC), autosomal recessive polycystic kidney disease (ARPKD), and Bardet-Biedl syndrome (BBS). Disease-causing variants in more than 50 genes have been identified to date. As the proteins encoded by almost all these genes localize to primary cilia and are organized in distinct functional modules (e.g., NPHP1-4–8 module), molecular processes linked to ciliary structure and/or function have been the pathophysiological focus of this heterogeneous disease group.

Despite major advances in molecular understanding, specific details of structural and functional ciliary protein interaction like that of NPHP1 and NPHP4, as well as the impact of individual genetic variants still need to be unraveled. Several in vitro models are available such as iPSCs, organoids, and 3D organoid cultures [1–3]. However, these approaches are time-consuming and depend on specialized laboratory settings making them unfeasible for routine clinical diagnostics [4–6].

Immunofluorescence (IF) analysis is a suitable diagnostic tool in the setting of primary cilia dyskinesia, a motile ciliopathy [7, 8]. Despite structural differences in the molecular composition of motile and non-motile cilia [9], the transition zone is similar in both cilia types. Previously, we have shown that NPHP1 localizes to the transition zone of both renal and respiratory cilia [10, 11]. Expression at respiratory cilia has also been reported for polycystin 1/2, the proteins altered in autosomal dominant polycystic kidney disease [12]. This prompted us to raise the question of whether immunofluorescence analysis (IF) using respiratory cilia carrying epithelial samples can also be used for the analysis of other NPHP proteins and the impact of disease-causing genetic variants on their ciliary localization.

Here, we aim to evaluate the usefulness of IF analysis of nasal epithelial cells to secure and accelerate the diagnosis of NPH. Additionally, we investigated the impact of disease-causing variants on NPHP protein localization in the setting of renal ciliopathies. We performed a large-scale IF screening with anti-NPHP1 and anti-NPHP4 antibodies using patient-derived respiratory epithelial cells from 111 individuals suffering from renal ciliopathies—85 of them with known genetic backgrounds.

## Methods

### Patient recruitment and nasal brushes

Individuals displaying a kidney phenotype genetically or clinically characterized as having renal ciliopathy were recruited via the Network of Early Onset Cystic Kidney Disease (NEOCYST) platform [13]. Patients were enrolled in the registry from February 1, 2010, through December 31, 2022. All patients and/or their parents gave written informed consent. The study was approved by the Ethics Committee of the University of Münster (2016–284-f-S) in accordance with the Declaration of Helsinki. Nasal brush biopsies were collected using protocols approved by the Institutional Ethics Review Board of the University of Münster (2016–284-f-S) and collaborating institutions as described previously [14].

### Genetic analyses

Owing to the registry-based multicenter study design, various methods for genetic testing were used, including PCR-based gel electrophoresis or MLPA for detection of homozygous *NPHP1* deletions, targeted Sanger sequencing on the basis of the patient’s phenotype, as well as multigene panel sequencing and exome sequencing as described previously [15]. Interpretation of the clinical significance of variants was completed following the American College of Medical Genetics and Genomics and the Association for Molecular Pathology guidelines [16] using the VarSome (VarSome The Human Genomics Community) online tool [17]. In addition to the automatically generated criteria, manual adjustment for the following 2 criteria was made: PM3 (recessive disorders, detected in trans with a pathogenic variant) and PP4 (patient’s phenotype or family history is highly specific for a disease with a single-genetic etiology). Further interpretation of functional effects of human nsSNPs was conducted using the PolyPhen-2 [18] (http://genetics.bwh.harvard.edu/pph2/), SIFT [19] (https://sift.bii.a-star.edu.sg/), PROVEAN [20] (http://provean.jcvi.org/about.php), varSEAK Online (https://varseak.bio, developed by JSI medical systems GmbH, Ettenheim, Germany), and ClinVar (release 13./31.07.2021) [21] (https://www.ncbi.nlm.nih.gov/clinvar/) online tools.

### Immunofluorescence analysis

Human respiratory epithelial cells (hRECs) were obtained from the middle turbinate by nasal brush biopsy and suspended in RPMI medium as described before [14].

For IF, hRECs were fixed using PFA or ice-cold methanol and blocked with skim milk or IgG-free BSA depending on the antibodies used.

The following primary antibodies were used: monoclonal mouse anti-acetylated α-tubulin (Sigma Aldrich, T6793), polyclonal rabbit anti-NPHP4 (Atlas Antibodies, HPA065526), and polyclonal rabbit anti-RSPH4A (Atlas Antibodies, HPA031196). Monoclonal mouse anti-NPHP1 (inhouse) and polyclonal rabbit anti-DNAH5 (inhouse) were reported previously [11]. The following secondary antibodies were used: goat-anti-rabbit Alexa Fluor 546 (Invitrogen, A11035), goat-anti-mouse Alexa Fluor 546 (Invitrogen, A11030), goat-anti-mouse Alexa Fluor Plus 488 (Invitrogen, A48286), and goat-anti-rabbit Alexa Fluor Plus 488 (Thermo Fisher Scientific, A48282). Nuclei were stained with Hoechst33342 (Thermo Fisher Scientific). IF images were taken using a Zeiss Apotome Axiovert 200 and a Zeiss LSM 880 Laser Scanning Microscope. Image processing was performed using AxioVersion 4.8. and ZEN-blue software. Adobe Illustrator was used for the final image processing.

To quantify the fluorescence of the stained proteins in the transition zone, equidistant intersecting planes for profile analysis were applied perpendicular to the maximum intensity line of the arc-shaped structure of the ciliary transition zone as described previously [22].

### Cell culture of primary respiratory epithelial cells on air–liquid interface (ALI)

hRECs for ALI culture were expanded on collagen-coated tissue flasks. Upon confluence, hRECs were subsequently transferred onto ALI inserts (CostarTM Corning® 3470 TranswellTM Clear Polyester Membrane Inserts for 24-well plates, Corning Incorporated) as described previously [23]. Cell cultures were expanded and maintained with PneumaCult EX-Medium and PneumaCult ALI-Medium according to manufacturers’ recommendations.

### Silver stain and western blot analyses

Lysates from hRECs were prepared from ALI cultures. Samples were lysed in NP40 buffer (50 mM Tris–Cl, pH 8.0, 500 mM NaCl, 1% IGEPAL, 0.5 mM EDTA, 10% glycerol). Dismembration was performed using a dismembrator (2000 rpm for 3 min).

Protein concentration was determined using the Qubit Protein Assay Kit (Thermo Fisher Scientific, Germany) to ensure equal loading quantities for silver stain and western blot analyses. Loading buffer was added (for each 100 µl of lysate: 50 µl 4 × NuPAGE LDS sample buffer, 30 µl Millipore-H2O, and 20 µl 1 M 1,4-dithiothreitol (DTT)). Silver staining was conducted using the Sigma ProteoSilver™ Silver Stain Kit.

Western blotting was performed as described previously [23] using NuPAGE 4–12% Bis–Tris gels and SeeBlue Plus2 as a marker. Electrophoresis was performed using a voltage flow with 200 V and 130 mA. Proteins were transferred to PVDF filters (Invitrogen) using transfer buffer (760 µl of ddH2O, 40 µl of 20 × transfer buffer). Incubation of the primary antibody was performed overnight at 4 °C. Primary antibodies against acetylated α-tubulin, NPHP1, and NPHP4 were provided as described above. Polyclonal rabbit anti-GAPDH (#2118) was purchased from Cell Signalling Technology.

Filters were incubated with horseradish peroxidase (HRP)-conjugated secondary antibody at room temperature before processing for enhanced chemiluminescence with ECL Prime Western Blotting reagents (GE Healthcare). Images were digitally acquired with a Peqlab FUSION-SL Advance Imager (VWR International); densitometry of western blots was carried out using Fiji software.

### Ca^2+^-dependent deciliation

Ca^2+^-dependent deciliation was performed as described previously [11]. In brief, inserts from ALI cultures were washed to remove mucus and cell debris using a deciliation buffer that contained 79.1 mM NaCl, 1.56 mM EDTA, 15.7 mM Tris–Cl (pH 7.5), 0.1% Triton-X 100, and 15.8 mM CaCl_2_. Inserts were gently scraped with a pipet tip to detach cells. Centrifugation (300 × g, 5 min) allowed the separation of deciliated cells from loose cilia. Cilia were collected from the supernatant by centrifugation (16,000 × g, 5 min). RPMI medium was added to the cell and cilia pellets for resuspension. The suspension was dripped out onto slides which were left to dry overnight and then frozen at − 20 °C until their use for IF.

## Results

### Co-localization of NPHP1 and NPHP4 at the transition zone of respiratory cilia in healthy controls

**Fig. 1:**
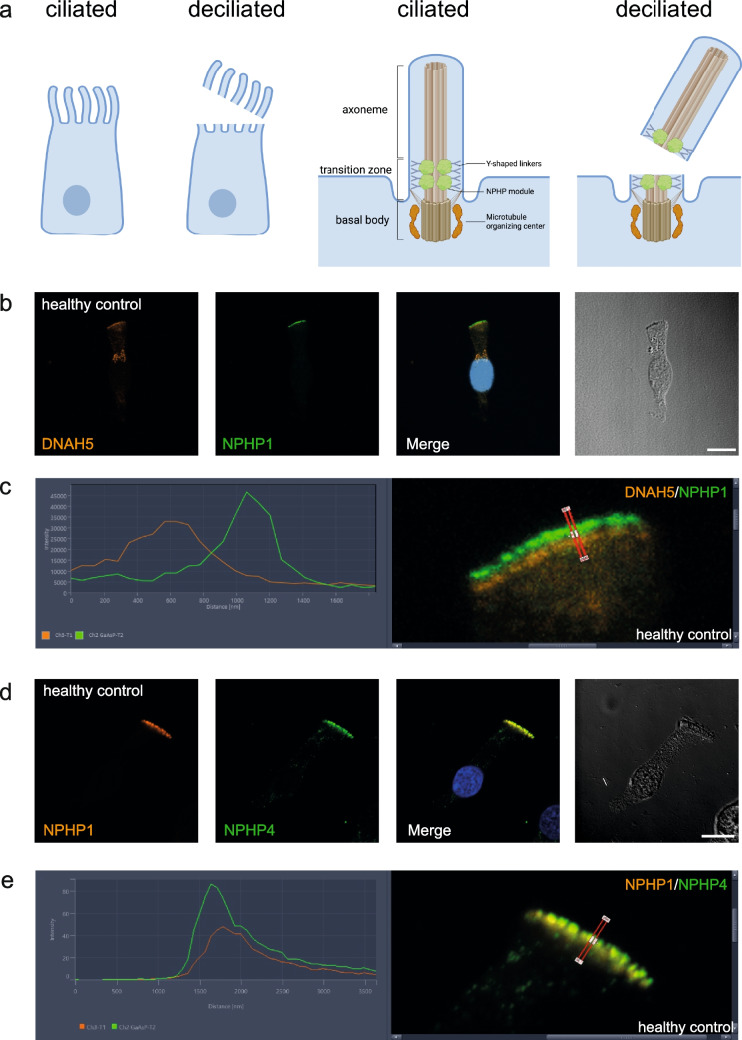
NPHP1 and NPHP4 co-localize at the ciliary transition zone in respiratory epithelial cells. **a** Schematic illustration of respiratory epithelial cells before and after deciliation. NPHP1 and NPHP4 (green) specifically localize to the transition zone, distal to the microtubule organizing center (MTOC, red) containing DNAH5. Upon deciliation, the cilia break off in the middle of the transition zone. Figure created with BioRender.com. **b**–**e** High-resolution immunofluorescence analysis of deciliated respiratory epithelial cells from healthy control individuals cultured under air–liquid interface (ALI) conditions. **b** Cells were stained with antibodies directed against DNAH5 (red) and NPHP1 (green). **c** Profiles of immunofluorescence intensity show a proximal staining of DNAH5 and a distal staining of NPHP1 with no overlap. **d** Cells were stained with antibodies directed against NPHP1 (red) and NPHP4 (green). NPHP1 and NPHP4 specifically co-localize at the transition zone. **e** Profiles of immunofluorescence intensity show an overlap of NPHP1 and NPHP4. Nuclei were stained with Hoechst33342 (blue). Scale bars represent 10 µm

To confirm that NPHP1 localizes distal to the microtubule organizing center (MTOC) of motile cilia, we performed IF analysis of DNAH5, an essential component of the microtubule organizing center, and NPHP1 on deciliated respiratory epithelial cells from ALI inserts of healthy controls (Fig. 1 a–c). During the process of deciliation, a rupture in the transition zone occurs, leaving half of the transition zone at the apical membrane of the cell and the other half at the base of the loose cilia (Fig. 1a).

Co-staining of deciliated respiratory epithelial cells using mouse monoclonal NPHP1 antibodies and rabbit polyclonal NPHP4 antibodies demonstrated a full overlap of IF signals (Fig. 1d). This was verified by corresponding densitometry results (Fig. 1e), proving that both NPHP1 and NPHP4 colocalize to the transition zone in respiratory epithelial cells.

### Impact of various ciliary gene variants on the localization of NPHP1

To evaluate the impact of different renal ciliopathy-causing gene variants on the protein composition of the transition zone and, in particular, the localization of NPHP1, we performed a large-scale IF screening of patient-derived respiratory epithelial cells applying anti-NPHP1 and anti-NPHP4 antibodies. Samples of 111 individuals were available, displaying one of the following renal ciliopathy phenotypes: polycystic kidney disease (ARPKD/ADPKD), nephronophthisis (NPH) or nephronophthisis related ciliopathies (NPH-RC), Bardet-Biedl Syndrome (BBS), and HNF1B nephropathy (HNF1B). In 84 cases, the diagnosis was genetically confirmed at the beginning of the study; 27 cases were molecularly unsolved despite a convincing phenotypic presentation—two of them being solved during the study. Homozygous or compound heterozygous disease-causing variants at the start of the study were distributed as follows: *NPHP1* (*n* = 11), *NPHP3* (*n* = 1), *NPHP4* (*n* = 1), *NPHP5/IQB1* (*n* = 1), *NPHP6/CEP290* (*n* = 5), *NPHP9/NEK8* (*n* = 1), *NPHP11/TMEM67* (*n* = 6), *NPHP13/WDR19* (*n* = 1), *IFT140* (*n* = 3), *MKS1* (*n* = 1), *PKHD1* (*n* = 7), and various *BBS* genes (*n* = 29). Heterozygous disease-causing variants for renal ciliopathies following a dominant trait were identified in *HNF1B* (*n* = 15) and *PKD1* (*n* = 2).

**Fig. 2:**
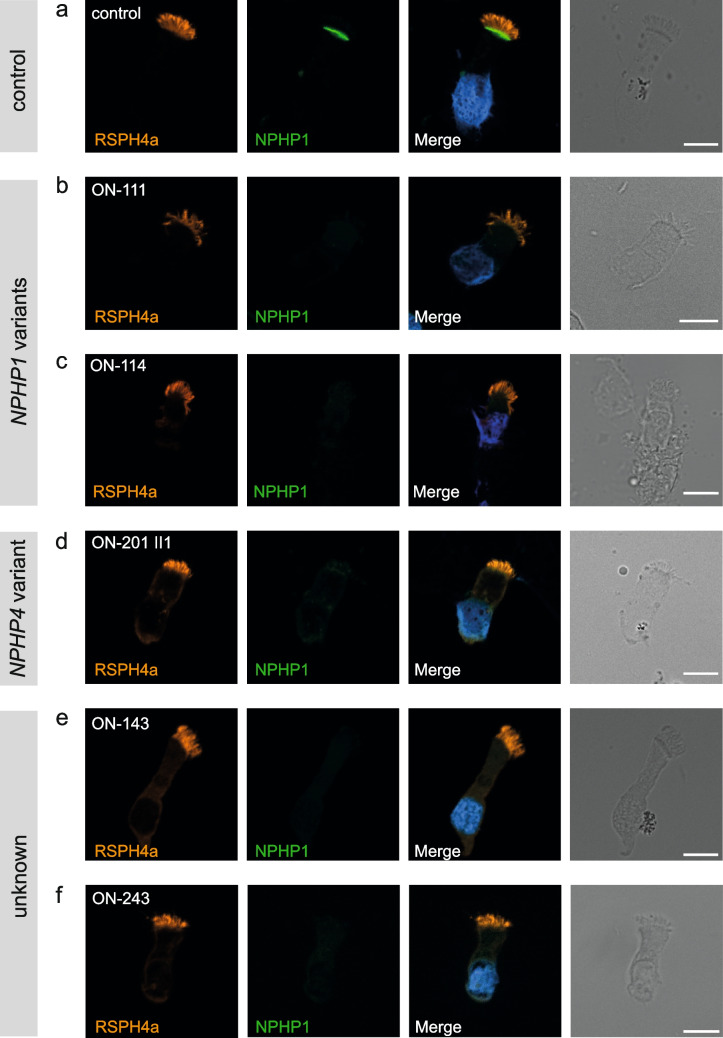
NPHP1 is absent from the ciliary transition zone of respiratory cells in individuals with pathogenic variants in *NPHP1* and *NPHP4*. High-resolution immunofluorescence analysis of respiratory epithelial cells from healthy control individuals and individuals carrying pathogenic variants in *NPHP1* and *NPHP4*. Cells were co-stained with antibodies against RSPH4a (ciliary marker, red) and NPHP1 (green). In control cells, NPHP1 localizes to the transition zone (**a**), whereas NPHP1 was absent in cells of individuals ON-111 (homozygous deletion of *NPHP1*), ON-243 (homozygous deletion of *NPHP1*), ON-114 (homozygous variant in *NPHP1*: c.1027G > A; p.Gly343Arg), ON-201 II1 (homozygous variant in *NPHP4*: c.3272del; p.Val1091fs), and ON-143 (homozygous variant in *NPHP4*: c.811-2144del; deletion Ex8-16). Nuclei were stained with Hoechst33342 (blue). Scale bars represent 10 µm

A total of 75/111 samples showed normal localization of NPHP1 comparable to healthy controls (Fig. 2a). In 14/111 samples, a complete absence of NPHP1 was observed. Twenty-two samples could not be evaluated due to the poor quality of nasal brush biopsy (*PKHD1* (*n* = 2), *BBS* (*n* = 10), *IFT140* (*n* = 2), *HNF1B* (*n* = 4), and unclear (*n* = 4)).

A total of 11/14 individuals showing a complete lack of NPHP1 signal displayed a pathogenic *NPHP1* genotype (Table 1), either in the form of a homozygous deletion of the entire *NPHP1* gene (*n* = 10) or the presence of a homozygous missense variant (c.1027G > A; p.Gly343Arg) (Fig. 2 b, c). The latter is of particular interest since this variant had been classified as a “variant of unknown significance” at the time, and we could now prove by IF staining that it is pathogenic.

**Table 1:** Genetic information for investigated individuals. The following reference sequences were used: NM_000272.5 for *NPHP1* and NM_015102.5 for *NPHP4*

Patient ID	Gene affected	DNA	Protein level	Allele 1	Allele 2	Variant type	ACMG-classification	Clinical phenotype
ON-111	*NPHP1*	Complete gene deletion	-	LOF	LOF	Deletion	Pathogenic	Congenital ocular motor apraxia (COMA)
ON-113	*NPHP1*	Complete gene deletion	-	LOF	LOF	Deletion	Pathogenic	Congenital ocular motor apraxia (COMA), Joubert syndrome
ON-114	*NPHP1*	c.1027G > A	p.Gly343Arg	MIS	MIS	Missense	Pathogenic	Isolated nephronophthisis
ON-116	*NPHP1*	Complete gene deletion	-	LOF	LOF	Deletion	Pathogenic	Isolated nephronophthisis
ON-119	*NPHP1*	Complete gene deletion	-	LOF	LOF	Deletion	Pathogenic	Congenital ocular motor apraxia (COMA = Cogan II)
ON-135	*NPHP1*	Complete gene deletion	-	LOF	LOF	Deletion	Pathogenic	Isolated nephronophthisis
ON-136	*NPHP1*	Complete gene deletion	-	LOF	LOF	Deletion	Pathogenic	Isolated nephronophthisis
ON-146	*NPHP1*	Complete gene deletion	-	LOF	LOF	Deletion	Pathogenic	Isolated nephronophthisis
ON-169	*NPHP1*	Complete gene deletion	-	LOF	LOF	Deletion	Pathogenic	Isolated nephronophthisis
ON-193 II1	*NPHP1*	Complete gene deletion	-	LOF	LOF	Deletion	Pathogenic	Joubert syndrome
ON-211 II1	*NPHP1*	Complete gene deletion	-	LOF	LOF	Deletion	Pathogenic	Senior-Løken syndrome
ON-243	*NPHP1*	Complete gene deletion	-	LOF	LOF	Deletion	Pathogenic	Senior-Løken syndrome
ON-201 II1	*NPHP4*	c.3272del	p.Val1091fs	LOF	LOF	Frameshift	Pathogenic	Isolated nephronophthisis
ON-143	*NPHP4*	c.811-2144del	deletion Ex8-16	LOF	LOF	Deletion	Pathogenic	Isolated nephronophthisis

*LOF* loss of function, *MIS* missense

A complete loss of the NPHP1 signal was also detected in the scenario of a pathogenic *NPHP4* genotype as presented by individual ON-201 II1 carrying a homozygous *NPHP4* frameshift mutation (c.3272del; p.Val1091fs) and clinically displaying an isolated NPH phenotype (Fig. 2d). For the two remaining individuals with no NPHP1 signal at the transition zone, a molecular diagnosis was not available from the clinical NEOCYST registry. However, the phenotypes reported for these two individuals were the Senior-Løken-syndrome with NPH in one case (ON-243, Fig. 2e) and isolated NPH in the other (ON-143, Fig. 2f). Objection of the staining results by the calculation of a signal-to-noise ratio was impossible for all 14 individuals owing to a complete lack of any NPHP1-related peak in the maximum intensity line—confirming a complete absence of nephrocystin 1.

### Impact of various ciliary gene variants on the localization of NPHP4

To determine the impact of *NPHP* and other disease-causing gene variants on the ciliary expression of NPHP4, we applied anti-NPHP4 antibody to respiratory cilia cell samples of 35 patients. Included were all individuals with an *NPHP* genotype (*NPHP1* (*n* = 11), *NPHP3* (*n* = 1), *NPHP4* (*n* = 1), *NPHP5/IQB1* (*n* = 1), *NPHP6/CEP290* (*n* = 5), *NPHP9/NEK8* (*n* = 1), *NPHP11/TMEM67* (*n* = 6), *NPHP13/WDR19* (*n* = 1)); one representative for each other available genetic entity (*PKHD1*, *MKS1*, *PKD1*, *HF1B*, *BBS*, *IFT140*); and the two genetically unsolved individuals where we observed a complete absence of NPHP1 (ON-243, ON-143).

**Fig. 3:**
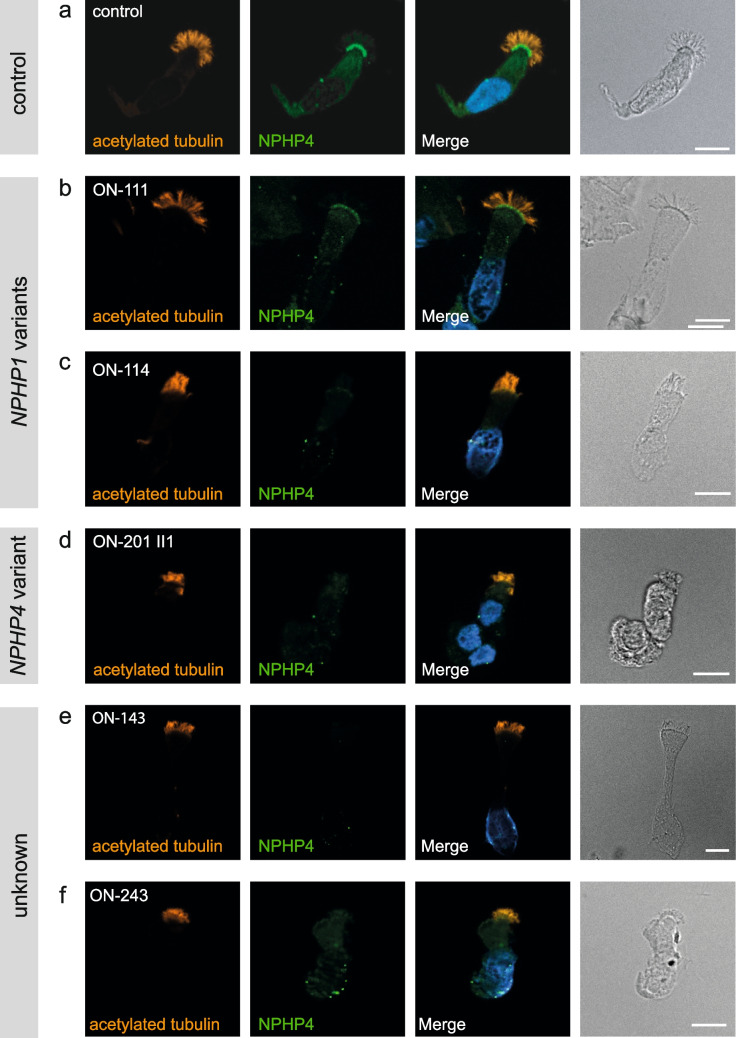
NPHP4 is severely reduced or absent from the transition zone of respiratory cells in individuals with pathogenic variants in *NPHP1* and *NPHP4*. High-resolution immunofluorescence analysis of respiratory epithelial cells from healthy control individuals and individuals carrying variants in *NPHP1* and *NPHP4*. Cells were co-stained with antibodies against acetylated-α-tubulin (ciliary marker, red) and NPHP4 (green). In control cells, NPHP4 localizes to the transition zone (**a**), whereas NPHP4 was severely reduced or absent in cells of individuals ON-111 (homozygous deletion of *NPHP1*), ON-243 (homozygous deletion of *NPHP1*), ON-201 II1 (homozygous variant in *NPHP4*: c.3272del; p.Val1091fs), and ON-143 (homozygous variant in *NPHP4*: c.811-2144del; deletion Ex8-16). Nuclei were stained with Hoechst33342 (blue). Scale bars represent 10 µm

NPHP4 clearly localized to the ciliary transition zone in both healthy controls (Fig. 3a) and the majority of renal ciliopathy patients (Supplementary Figs. 3 and 4). A complete axonemal absence of NPHP4 was observed in individual ON-201 II1, who was molecularly characterized by a homozygous disease-causing *NPHP4* loss of function variant (Fig. 3b). All 11 patients with an *NPHP1* genotype displayed a clearly altered NPHP4 IF signal compared to healthy controls with either severely reduced signal intensity or complete loss of signal at the transition zone (ON-111; Fig. 3c). This also applied to individual ON-114 carrying the homozygous *NPHP1* missense variant as described above (c.1027G > A; p.Gly343Arg). To objectify this impression, a signal-to-noise ratio (SRN) was calculated based on the NPHP4 intensity in the transition zone and the background signal in the cytoplasm. This revealed a mean value of 9.21 for healthy controls, whereas the mean SNR in individuals carrying NPHP1 variants was 2.57 (*p* = 0.008). The two genetically unsolved patients displayed an altered NPHP4 IF pattern as well: A complete absence of the NPHP4 signal was observed for ON-143 (Fig. 3e), and the IF staining of the ON-243 samples revealed a severely reduced signal as observed for the NPHP1 cohort before (Fig. 3d). 1/35 samples could not be evaluated due to the poor quality of nasal brush biopsy (*NPHP6/CEP290*).

### Extended genetic analysis

**Fig. 4:**
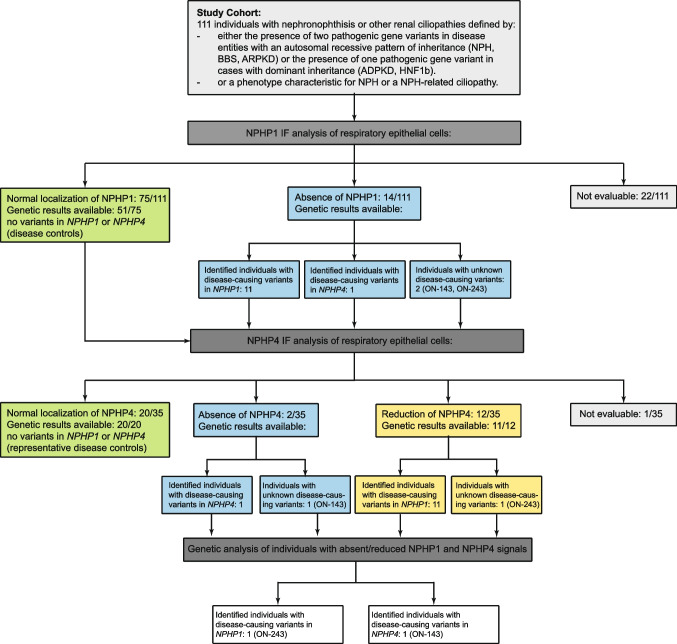
Flow chart displaying an overview of the results of subsequent NPHP1/NPHP4 immunofluorescence and genetic analyses of 111 individuals with renal ciliopathies

Extended genetic analysis was applied to the genetically unsolved patients with the clearly altered IF results comprising whole exome sequencing (WES) and MLPA analysis. We were able to identify a homozygous *NPHP1* deletion in individual ON-243 and a pathogenic homozygous deletion resulting in a frameshift of *NPHP4* in individual ON-143 (c.811-2144del; deletion Ex8-16) (Fig. 4).

### Western blot

**Fig. 5:**
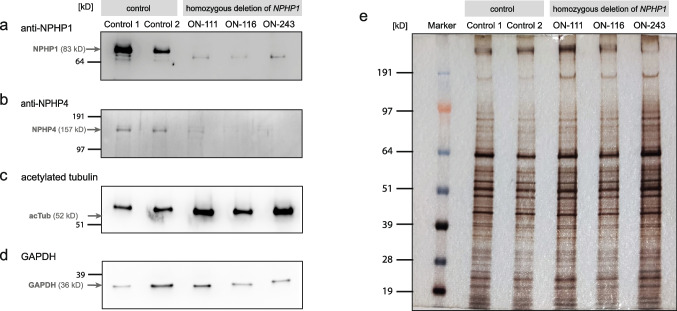
Western blot analysis of respiratory epithelial cells from individuals with pathogenic variants in *NPHP1* demonstrate the absence of NPHP1 and the reduction of NPHP4. Immunoblotting of whole-cell lysates from respiratory epithelial cells after air–liquid interface (ALI) culture. **a** Immunoblotting with anti-NPHP1 demonstrates the detection of the 83 kDa large isoform of NPHP1 (O15259-1) in the control. In individuals ON-111, ON-116, and ON-243, carrying variants in *NPHP1*, this band was absent. The subtle smaller bands detectable in all lanes represent an unspecific binding of the antibody. **b** Immunoblotting with anti-NPHP4 demonstrates the detection of the 157 kDa large isoform of NPHP4 (O75161-1) in the control. In individuals ON-111 (homozygous deletion of *NPHP1*), ON-116 (homozygous deletion of *NPHP1*), and ON-243 (homozygous deletion of *NPHP1*), this band was either absent or severely reduced. The subtle higher bands detectable in all lanes represent an unspecific binding of the antibody. **c** Immunoblotting with anti-acetylated-α-tubulin confirmed equal amounts of ciliary proteins within the different lysates. **d** Immunoblotting with anti-GAPDH confirmed equal protein concentrations of the whole-cell lysates. **e** Silver staining demonstrates the integrity of protein recovery from the whole cell preparation

To rule out IF staining-associated artifacts, western blot analysis was performed using whole cell lysates from respiratory epithelial cells after ALI culture from representative samples with *NPHP1* deletion (ON-111, ON-116, ON-243) as well as healthy controls. No cell lysates were available from NPHP4 patients. While in healthy controls, a clear band of the 83 kDa isoform of NPHP1 was observed, no NPHP1 band could be detected in any of the patient lysates (Fig. 5a). Densitometry of the western blot confirmed no detectable signal in all lanes of the patient lysates. The same applied to NPHP4: while healthy controls displayed a clear band of the 157 kDa NPHP4 protein, in patient lysates, the NPHP4 bands were severely reduced when normalized to control (ON-111 24.1%; ON-116 32.2%; ON-243 26.4%) (Fig. 5b). Western blots using an anti-acetylated α-tubulin antibody (Fig. 5c) and an anti-GAPDH antibody (Fig. 5d) revealed comparable protein concentrations within the lysates. Comparable loading of all samples was also confirmed by silver staining (Fig. 5e).

## Discussion

Despite the large number of variants identified in 23 NPHP genes to date, up to 40% of NPH patients remain genetically unsolved [24]. In some cases with convincing phenotype, only one heterogeneous gene defect is found even when applying state-of-the-art sequencing techniques. Many reported variants are classified as a “variant of unknown significance” and cannot be regarded as “disease-causing” with absolute certainty. Different approaches have been used in order to tackle this challenge with varying degrees of success [25, 26] risking that genetic results remain inconclusive.

Immunofluorescence (IF) staining of respiratory epithelial cells offers a technique to verify the pathogenic impact of inconclusive genetic results confirming the clinical diagnosis. Respiratory epithelial cells are obtained via non-invasive nasal brush biopsy [14], are not impacted in their structure by kidney transplantation, and can be analyzed directly after sampling. In motile ciliopathies such as primary ciliary dyskinesia (PCD), IF analysis has already been proven to be a convenient diagnostic tool with high comfort and minimal invasiveness for the patient [13].

In this study, we applied an IF-based approach to a large cohort of 111 individuals with either nephronophthisis (*n* = 58) or other renal ciliopathies (*n* = 53) using antibodies directed against NPHP1 and NPHP4. We included both *NPHP1* and *NPHP4* null mutants proving the specificity of used antibodies as recommended by standard good practice guidelines [14].

The pathogenicity of all NPHP1 variants was verified by showing a complete absence of the NPHP1 IF signal. This included one individual carrying a homozygous *NPHP1* missense variant (c.1027G > A; p.Gly343Arg) formerly classified as a “variant of unknown significance” according to ACMG criteria (PP5, PP3, and PM2). Furthermore, the results revealed an absence of NPHP1 in individuals with an *NPHP4* genotype. Vice versa, NPHP4 expression at the ciliary transition zone was severely reduced or completely absent in individuals with an *NPHP1* genotype. A complete axonemal absence of NPHP4 was observed in the molecular scenario of a homozygous *NPHP4* loss of function variant proving the specificity of the antibody’s NPHP4 recognition at the transition zone. However, in the same individual, we observed some weak cytoplasmic signals. These were most probably explained by unspecific staining effects that are frequently observed in polyclonal antibody use (also variably present in other individuals, Fig. 3). According to the current state of knowledge, nephrocystins—the gene products of *NPHP* genes—are organized in at least four distinct functional modules: the NPHP1-4–8 module, NPHP2-3–9-ANKS6 module, NPHP5-6 module, and the MKS module. These nephrocystin modules are related to different signaling pathways, including the Wnt-, Hedgehog-, DNA damage response (DDR)-, Hippo-, intracellular calcium signaling-, cAMP signaling-, and mTOR pathway [27]. However, so far, these observations were obtained by cell culture-based functional interaction studies only. Our results verify this molecular relationship between NPHP1 and NPHP4 through abnormal protein expression in vivo.

Previously, the expression of other proteins associated with a renal ciliopathy phenotype like *CEP290/NPHP6* and polycystin1 and 2 has been reported at airway epithelia as well as at the olfactory bulb [28, 29]. We were able to reveal a significant olfactory deficit not only in BBS patients but also in individuals with a *TMEM67/NPHP11*-related phenotype [30]. Yet, despite the expression and an obvious interplay of nephrocystins at the transition zone, the molecular relevance of these proteins for motile cilia functional integrity largely remains unclear. Brndiarova et al. reported an altered motile cilia function in individuals affected by primary renal ciliopathies [31]. Furthermore, an increased prevalence of bronchiectasis has been observed in ADPKD patients, suggesting disturbed mucociliary clearance [12]. Further functional studies characterizing and quantifying ciliary motility and most of all standardized assessments of clinical airway-related symptoms will be needed to evaluate the functional impact of nephrocystins and polycystins on motile ciliary function.

BBS proteins and the transcription factor *HNF1B* also have a close functional relationship to cilia architecture and cilia-related transport processes. While BBS proteins—organized in a multimeric complex called BBSome—play a key role in cilia-related protein transport, *HNF1B* has been proven to directly regulate the expression of *PKHD1*, *PKD2*, and other genes causative for renal ciliopathies with a cystic phenotype [32]. In our study, we discovered that *BBS* and *HNF1B* genes do not have an impact on nephrocystin expression at the ciliary transition zone as IF results of all individuals with a genotype other than *NPHP1/4* including BBS (*n* = 29) and HNF1B (*n* = 15) did not show any effect on NPHP1 or NPHP4 expression.

Non-specific staining of basal bodies by polyclonal antibodies has previously been reported as problematic in IF analyses [14]. To address potential issues, we performed western blot analyses using lysates obtained from healthy controls and *NPHP1* individuals. Bands for NPHP1 and NPHP4 in lysates from healthy controls were specific and comparable in intensity while severely reduced or completely absent in *NPHP1* individuals. We congruently verified the absence or severe reduction of NPHP1 and NPHP4 by both techniques—IF staining and western blot analysis. Unfortunately, there were no cilia lysates available for western blot analysis from the two individuals with an NPHP4 genotype as those samples were submitted as slides instead of native cell culture material, making cell culture impossible.

Our study suffers from several limitations—above all the small NPHP4 sample size comprising only two individuals with a corresponding genotype. However, one must take into account that *NPHP4* variants make up only 3–7% of NPH cases overall [33, 34]. Another limitation was the relatively high number of poor-quality samples, particularly in the BBS cohort. There is no clear explanation for this observation. Bacterial contamination due to frequent respiratory infections seems to play a certain role. Yet, this assumption still must be objectively confirmed. Also, there might have been some kind of sampling error since the majority of BBS samples have been collected during BBS patient days which were held abroad. Despite these limitations, the results obtained in our study were congruent in both IF staining and western blots and underlined previous findings from functional studies regarding NPHP1 and NPHP4 interaction [8, 35, 36].

In our study, the IF-based approach was not only suitable to verify inconclusive genetic results but also could be applied as an efficient screening tool to streamline genetic analysis. This was exemplified by the two cases ON-143 and ON-243, which, at the beginning of the study, were registered as genetically unsolved despite a convincing phenotype for NPH and the Senior-Løken syndrome, respectively. IF results revealed a complete absence of NPHP1 and NPHP4 in one case (ON-243)—as observed in the *NPHP1* cohort—and a combination of absent NPHP4 with severely reduced NPHP1 in the other one (ON-143)—comparable to the pattern found in NPHP4 individuals—prompting us to apply a comprehensive genetic analysis. Strikingly, in both individuals, we were able to ascertain a genetic diagnosis matching their IF pattern: a homozygous *NPHP1* deletion in ON-243 and a homozygous deletion resulting in a frameshift of *NPHP4* (c.811-2144del; deletion Ex8-16) in ON-143. Thus, in cases presenting a coherent phenotype, preceding IF screening might help to stratify and limit genetical efforts.

Certainly, in the scenario of fast and low-cost genetic analyses, the IF-based approach neither has the potential nor was intended to compete with or even replace genetic diagnostics in renal ciliopathies. However, this method has the potential to act as an ideal complement to the current standard of genetic diagnostics particularly in cases with inconclusive genotypes. Beyond that, it can serve as a model system to investigate the molecular interplay of nephrocystins in human tissue rather than animal models.

## Conclusion

We were able to show that IF analysis of patient-derived respiratory epithelial cells is a helpful tool to secure and accelerate the diagnosis of nephronophthisis—both by verifying inconclusive genetic results and by stratifying genetic diagnostic approaches. For individuals carrying disease-causing variants in *NPHP1* and *NPHP4*, we defined distinctive IF patterns either characterized by the absence of both—NPHP1 and NPHP4—(*NPHP1* genotype) or the combination of severely reduced NPHP1 with absent NPHP4 (*NPHP4* genotype). In addition, we provide in vivo evidence for the hypothesis of the functional NPHP1-4–8 module that has been suggested based on functional interaction studies previously.

## NEOCYST consortium

C. Bergmann**,** Mainz, Germany; M. Cetiner, Essen, Germany; J. Drube**,** Hannover, Germany; C. Gimpel**,** Heidelberg, Germany; J. Göbel**,** Frankfurt, Germany; D. Haffner**,** Hannover, Germany; T. Illig**,** Hannover, Germany; N. Klopp**,** Hannover, Germany; J. König**,** Münster, Germany; M. Konrad, Münster, Germany; M. C. Liebau, Cologne, Germany; S. Lienkamp, Zürich, Germany; C. Okorn, Essen, Germany; H. Omran, Münster, Germany; L. Pape, Essen, Germany; P. Pennekamp, Münster, Germany; F. Schaefer, Heidelberg, Germany; B. Schermer, Cologne, Germany; H. Storf, Frankfurt, Germany; A. Titieni, Wien, Germany; Germany; S. Weber, Marburg, Germany; W. Ziegler, Hannover, Germany. I. Kamp-Becker, Marburg; J. Vasseur, Frankfurt, Germany; M. Dahmer-Heath, Münster, Germany; S. Kollmann, Münster, Germany. J. Gerß, Münster, Germany.

## Supplementary Information

Below is the link to the electronic supplementary material.

Graphical abstract (PPTX 772 KB)

Supplementary file2 (PDF 967 KB)

Supplementary file3 (PDF 1009 KB)

Supplementary file4 (PDF 1281 KB)

Supplementary file5 (PDF 1279 KB)

Supplementary file6 (PDF 234 KB)

## Data Availability

The datasets generated during and/or analyzed during the current study are available from the corresponding author on reasonable request.
