# Supplementary material for: Immunofluorescence analyses of respiratory epithelial cells aid the diagnosis of nephronophthisis
Source: Pediatr Nephrol. 2024 Aug 5;39(12):3471–83. doi: 10.1007/s00467-024-06443-0 (PMC11511759; doi:10.1007/s00467-024-06443-0)
Supplement: Supplementary file 1 — Graphical abstract (PPTX 772 KB) [file 467_2024_6443_MOESM1_ESM.pptx]

## Slide 1
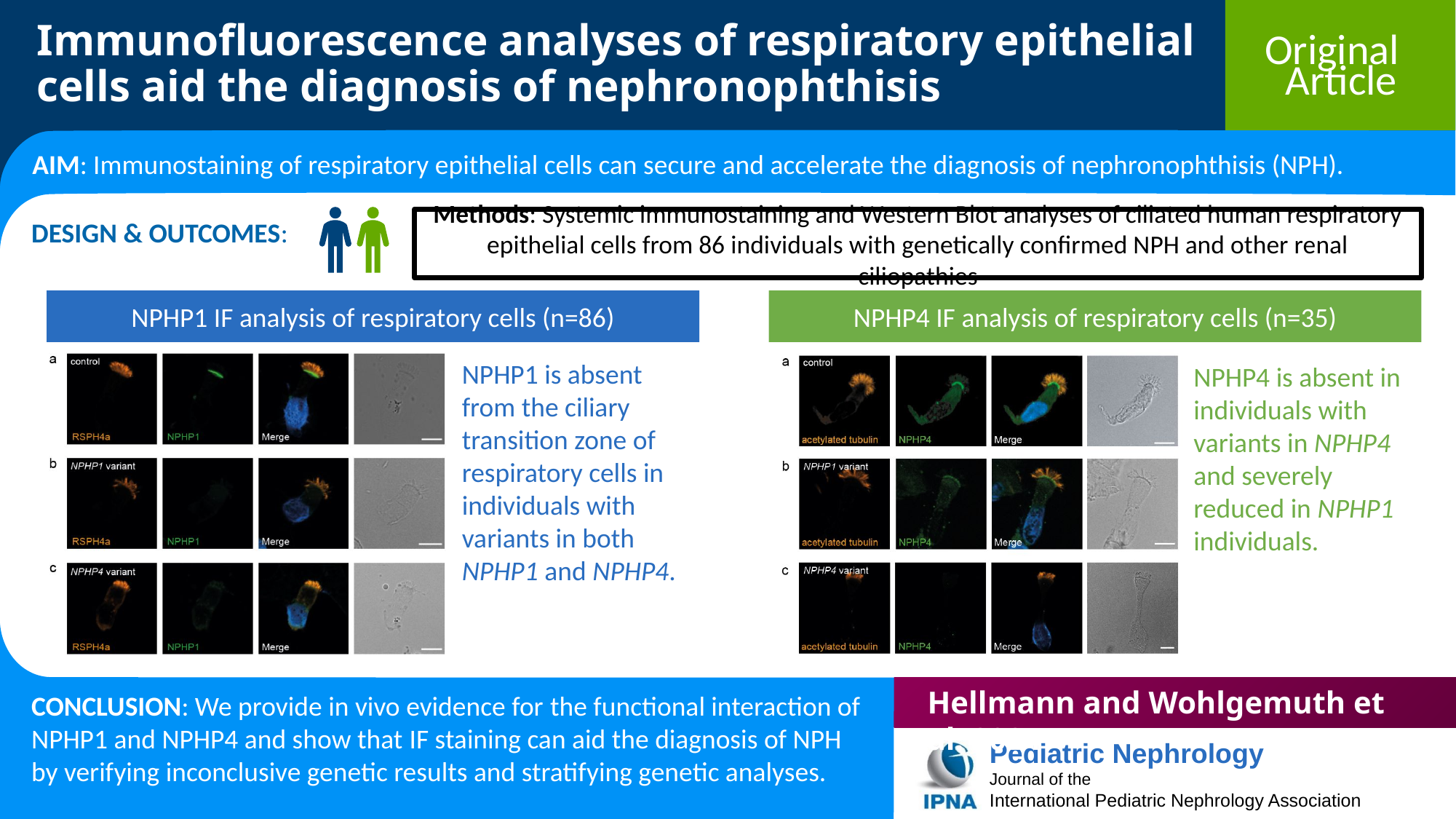

Immunofluorescence analyses of respiratory epithelial cells aid the diagnosis of nephronophthisis
AIM: Immunostaining of respiratory epithelial cells can secure and accelerate the diagnosis of nephronophthisis (NPH).
Methods: Systemic immunostaining and Western Blot analyses of ciliated human respiratory epithelial cells from 86 individuals with genetically confirmed NPH and other renal ciliopathies
DESIGN & OUTCOMES:
NPHP4 IF analysis of respiratory cells (n=35)
NPHP1 IF analysis of respiratory cells (n=86)
NPHP1 is absent from the ciliary transition zone of respiratory cells in individuals with variants in both NPHP1 and NPHP4.
NPHP4 is absent in individuals with variants in NPHP4 and severely reduced in NPHP1 individuals.
Hellmann and Wohlgemuth et al. 2024
CONCLUSION: We provide in vivo evidence for the functional interaction of NPHP1 and NPHP4 and show that IF staining can aid the diagnosis of NPH by verifying inconclusive genetic results and stratifying genetic analyses.
