# Supplementary figures and images for: Immunofluorescence analyses of respiratory epithelial cells aid the diagnosis of nephronophthisis
Source: Pediatr Nephrol. 2024 Aug 5;39(12):3471–83. doi: 10.1007/s00467-024-06443-0 (PMC11511759; doi:10.1007/s00467-024-06443-0)

Suppl. Fig. 1

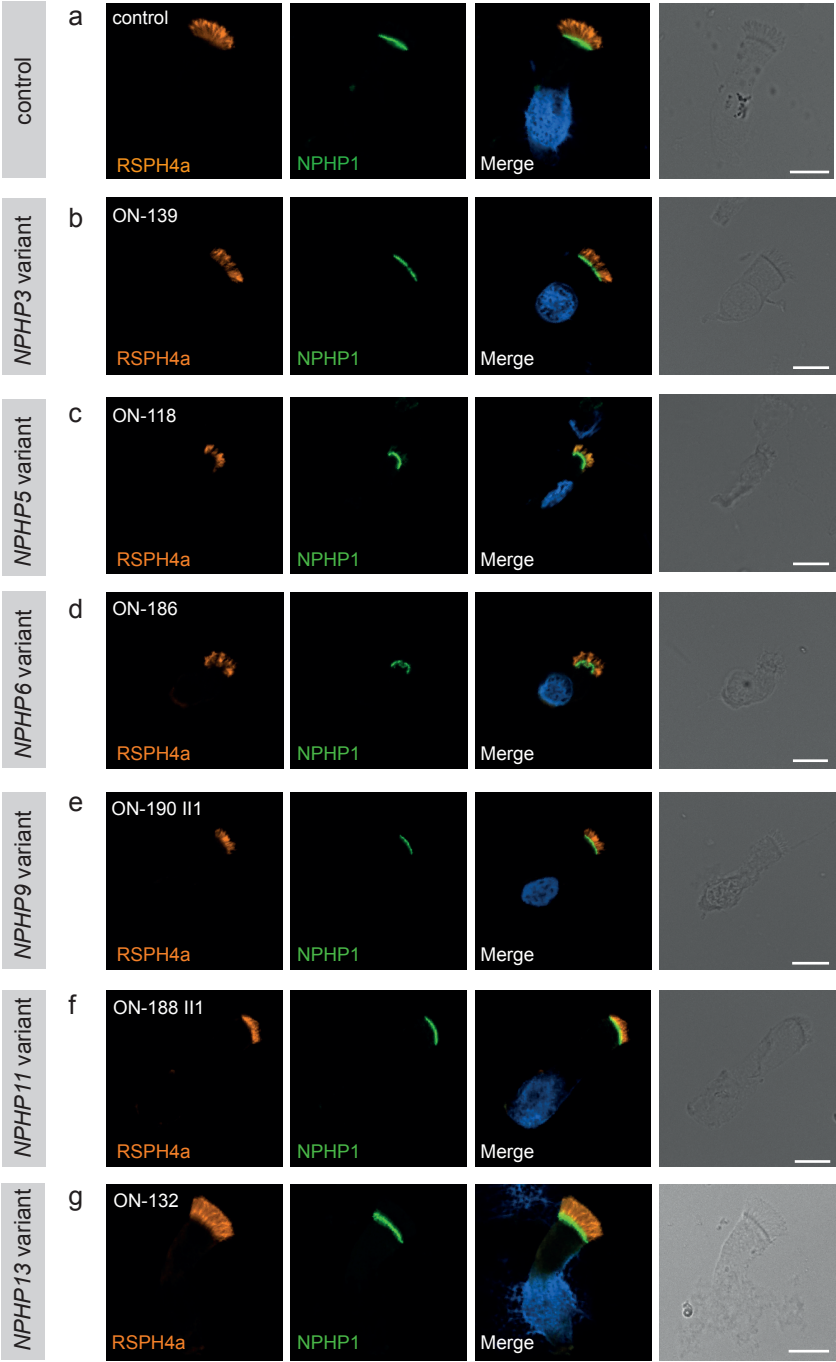

Supplement: Supplementary file 2 — Supplementary file2 (PDF 967 KB) [file 467_2024_6443_MOESM2_ESM.pdf]

Suppl. Fig. 2

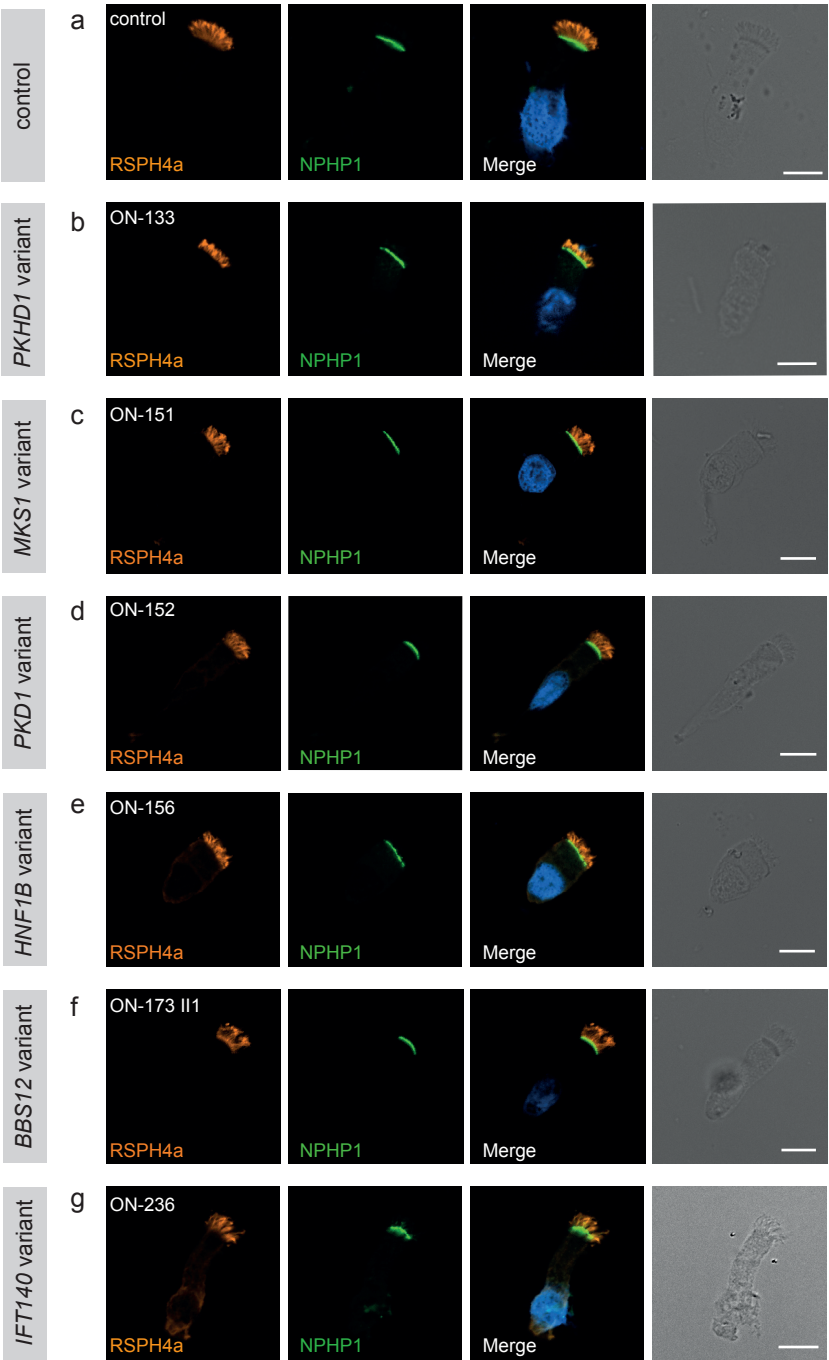

Supplement: Supplementary file 3 — Supplementary file3 (PDF 1009 KB) [file 467_2024_6443_MOESM3_ESM.pdf]

Suppl. Fig. 3

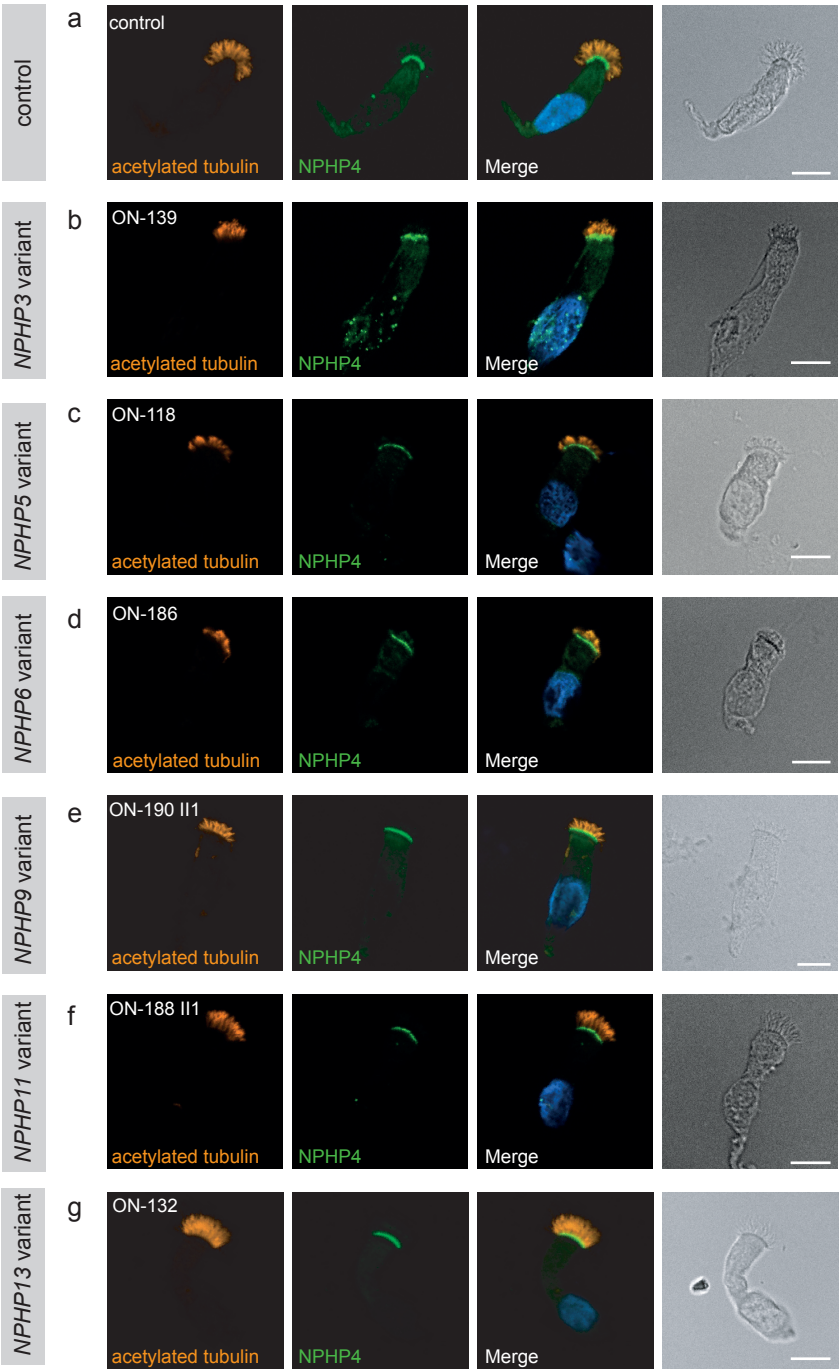

Supplement: Supplementary file 4 — Supplementary file4 (PDF 1281 KB) [file 467_2024_6443_MOESM4_ESM.pdf]

Suppl. Fig. 4

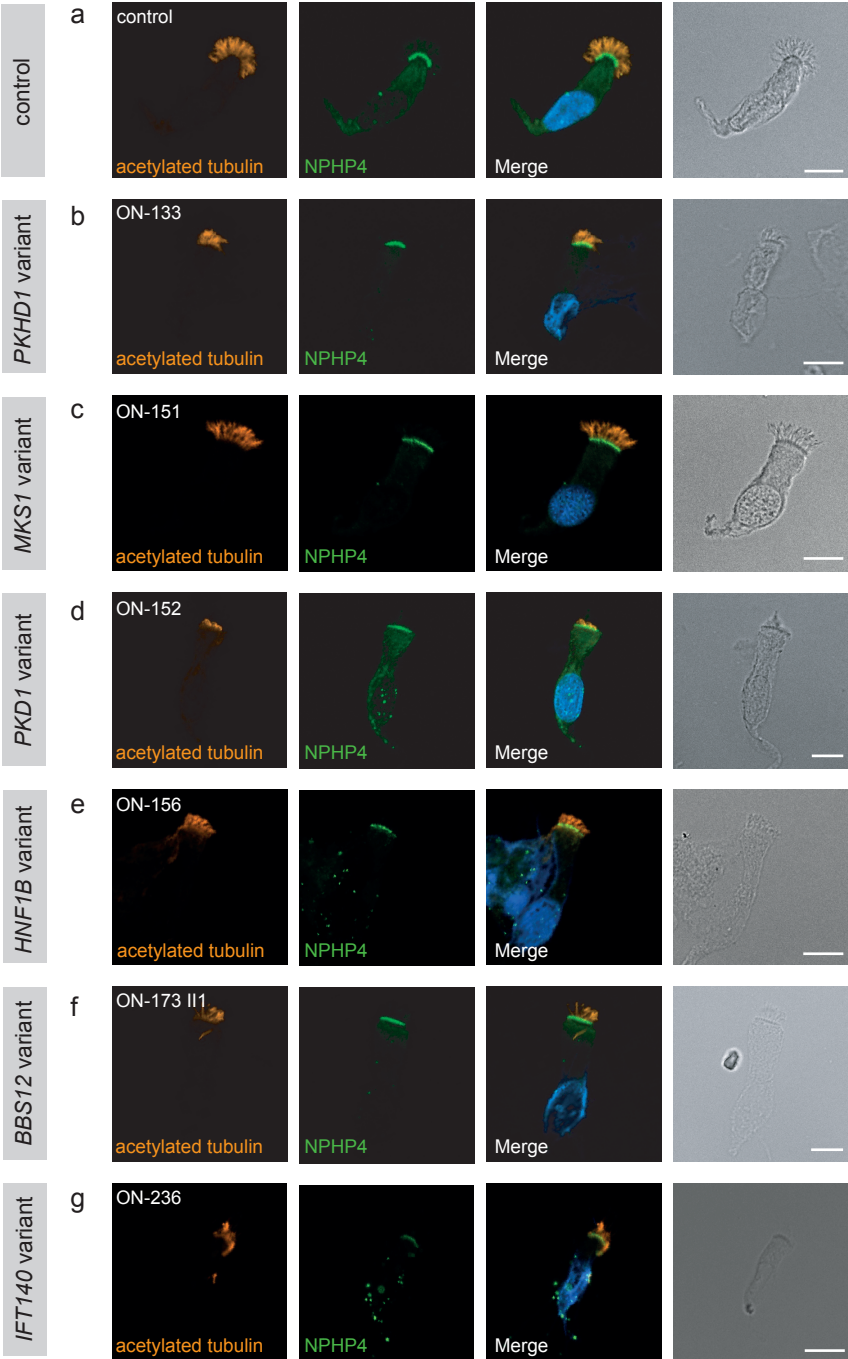

Supplement: Supplementary file 5 — Supplementary file5 (PDF 1279 KB) [file 467_2024_6443_MOESM5_ESM.pdf]
