# Supplementary material for: Immunofluorescence analyses of respiratory epithelial cells aid the diagnosis of nephronophthisis
Source: Pediatr Nephrol. 2024 Aug 5;39(12):3471–83. doi: 10.1007/s00467-024-06443-0 (PMC11511759; doi:10.1007/s00467-024-06443-0)
Supplement: Supplementary file 6 — Supplementary file6 (PDF 234 KB) [file 467_2024_6443_MOESM6_ESM.pdf]

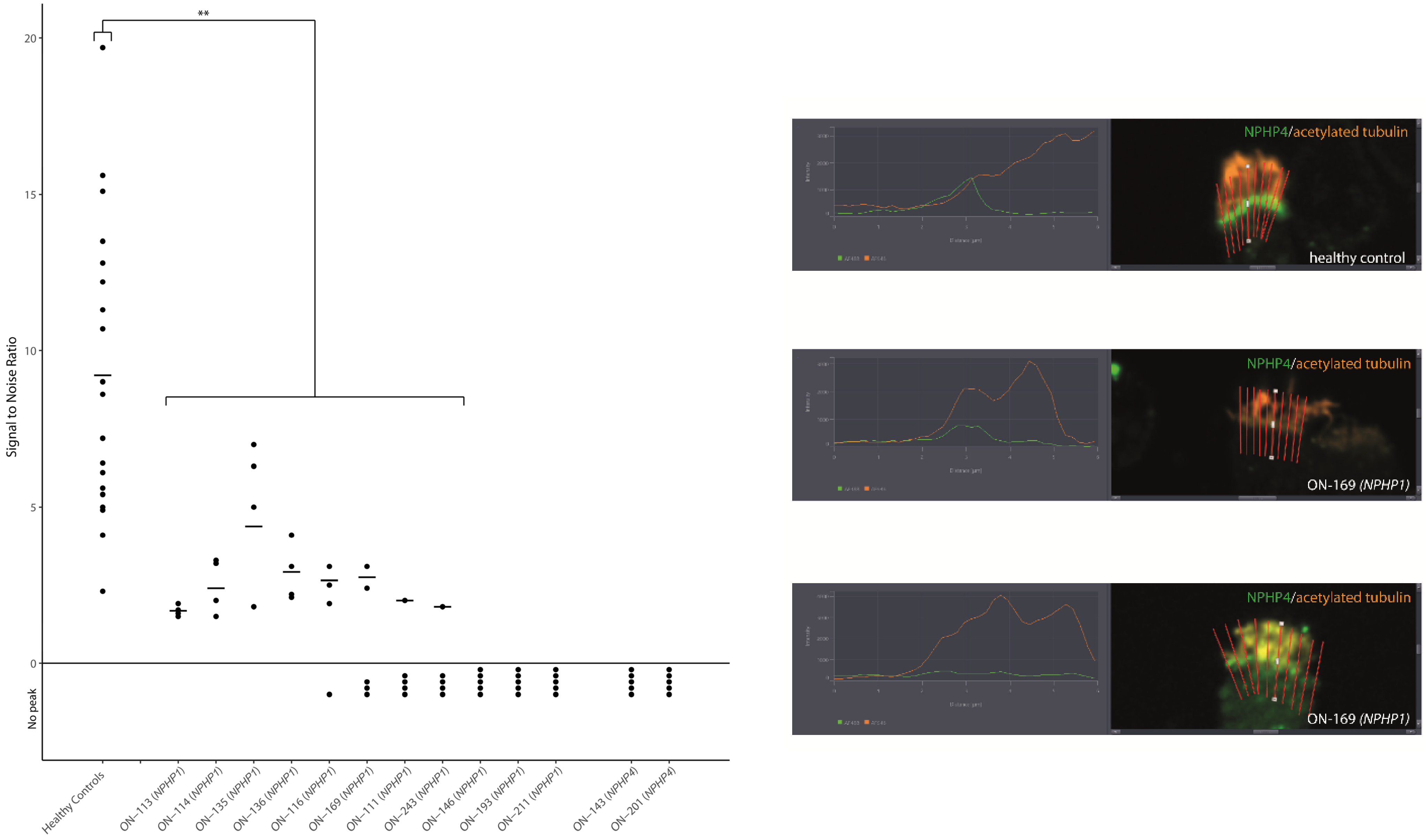
**
20
15
10
5
0
Signal to Noise Ratio
No peak
Healthy Controls
ON–113 (NPHP1)
ON–114 (NPHP1)
ON–135 (NPHP1)
ON–136 (NPHP1)
ON–116 (NPHP1)
ON–169 (NPHP1)
ON–111 (NPHP1)
ON–243 (NPHP1)
ON–146 (NPHP1)
ON–193 (NPHP1)
ON–211 (NPHP1)
ON–143 (NPHP4)
ON–201 (NPHP4)
NPHP4/acetylated tubulin
healthy control
NPHP4/acetylated tubulin
ON-169 (NPHP1)
NPHP4/acetylated tubulin
ON-169 (NPHP1)
